# The Cerebellum and SIDS: Disordered Breathing in a Mouse Model of Developmental Cerebellar Purkinje Cell Loss during Recovery from Hypercarbia

**DOI:** 10.3389/fneur.2016.00078

**Published:** 2016-05-13

**Authors:** Michele A. Calton, Jeremy R. Howard, Ronald M. Harper, Dan Goldowitz, Guy Mittleman

**Affiliations:** ^1^Department of Psychology, The University of Memphis, Memphis, TN, USA; ^2^Neurobiology, David Geffen School of Medicine, University of California, Los Angeles, Los Angeles, CA, USA; ^3^Centre for Molecular Medicine and Therapeutics, University of British Columbia, Vancouver, BC, Canada; ^4^Department of Psychological Science, Ball State University, Muncie, IN, USA

**Keywords:** cerebellum, Purkinje cells, disordered breathing, sudden death, SIDS, SUDC, SUDEP

## Abstract

The cerebellum assists coordination of somatomotor, respiratory, and autonomic actions. Purkinje cell alterations or loss appear in sudden infant death and sudden death in epilepsy victims, possibly contributing to the fatal event. We evaluated breathing patterns in 12 wild-type (WT) and *Lurcher* mutant mice with 100% developmental cerebellar Purkinje cell loss under baseline (room air), and recovery from hypercapnia, a concern in sudden death events. Six mutant and six WT mice were exposed to 4-min blocks of increasing CO_2_ (2, 4, 6, and 8%), separated by 4-min recovery intervals in room air. Breath-by-breath patterns, including depth of breathing and end-expiratory pause (EEP) durations during recovery, were recorded. No baseline genotypic differences emerged. However, during recovery, EEP durations significantly lengthened in mutants, compared to WT mice, following the relatively low levels of CO_2_ exposure. Additionally, mutant mice exhibited signs of post-sigh disordered breathing during recovery following each exposure. Developmental cerebellar Purkinje cell loss significantly affects compensatory breathing patterns following mild CO_2_ exposure, possibly by inhibiting recovery from elevated CO_2_. These data implicate cerebellar Purkinje cells in the ability to recover from hypercarbia, suggesting that neuropathologic changes or loss of these cells contribute to inadequate ventilatory recovery to increased environmental CO_2_. Multiple disorders, including sudden infant death syndrome (SIDS) and sudden unexpected death in epilepsy (SUDEP), appear to involve both cardiorespiratory failure and loss or injury to cerebellar Purkinje cells; the findings support the concept that such neuropathology may precede and exert a prominent role in these fatal events.

## Introduction

Sudden unexpected death occurs in several syndromes, with mechanisms underlying the fatal event not yet understood. Sudden infant death syndrome (SIDS) is one such syndrome; the condition occurs in infants <1 year of age, often during sleep, and with victims succumbing suddenly with major causes excluded even after autopsy and a thorough investigation (1, 2). Sudden death in epilepsy (SUDEP), appears in adults as well as older children, often occurs during sleep, but leaves few signs of mechanisms operating to induce a fatal event. A defect in breathing, or deficient responses to a ventilatory challenge is suspected in both SIDS and SUDEP. Although an inability to respond to severe, transient hypotension is a strong possibility (3, 4), the circumstances surrounding both SIDS and SUDEP conditions suggest an unknown brain abnormality that results in an inability to appropriately compensate for, or recover from, exposure to an intrinsic or exogenous stressor (5). Here, we evaluate the role of the cerebellum in mediating the consequences of exposure to one exogenous stressor, increased levels of environmental carbon dioxide (CO_2_; hypercapnia).

The focus on high CO_2_ arises from the circumstances normally associated with SIDS or postictal cessation of breathing in epilepsy. SIDS and SUDEP victims are often found deceased in bed, in the prone position, and sometimes in soft bedding, focusing attention on exposure to elevated CO_2_ concentrations associated with these conditions during sleep (6, 7). Thus, SIDS likely results from an inability to respond appropriately with respiratory and cardiovascular patterns to accommodate such a breathing stressor. Similarly, postictal respiratory failure in SUDEP would elevate CO_2_ levels, imposing a breathing challenge that brain structures may not be able to manage.

Structures within the cerebellum play a significant role in responding to extreme respiratory or blood pressure challenge (8–11). Developmental neuropathology of the cerebellum, as well as surgical or other insult to this structure, is accompanied by various forms of disordered breathing, including apnea, obstructive apnea, and hypoventilation (12–16). Such disordered breathing induces substantial increases in markers for CO_2_ (17), which, if not compensated, could place the subject at risk. Cerebellar neuropathology appears in SIDS victims, with a higher incidence of delayed cerebellar cortex maturation (18), and increased Purkinje cell apoptosis than controls (19). Cerebellar injury is very common in patients with epilepsy, likely induced either by excitotoxic injury or as a consequence of long-term antiepileptic medication. Additionally, in SIDS, transcriptional nucleolar activity is significantly reduced in cerebellar Purkinje cells relative to controls, suggesting hypofunction of the Purkinje cells in these victims. The nature of mechanisms underlying these pathologies or whether they underlie failure is unknown (20).

Cerebellar Purkinje cells provide the sole output of the cerebellar cortex, and innervate the deep cerebellar nuclei which, in turn, send efferents to multiple areas of the brainstem involved in respiratory and autonomic functions (21, 22). One pair of nuclei, the fastigial nuclei (FN), is responsive to chemical respiratory challenges, with cell body lesions of these nuclei significantly attenuating compensatory respiratory responses to increased levels of environmental CO_2._ However, such lesions have little effect on relaxed, rhythmic breathing (10, 23). Additionally, *Lurcher* mutant mice (*Lc/*+) with global, developmental cerebellar Purkinje cell loss show reduced respiratory responses following exposure to increased environmental CO_2_ (24), an outcome interpreted as a reduction in sensitivity to increased blood CO_2_ levels (25–27).

Exposure to increased CO_2_ in the environment is commonly encountered in everyday life, resulting in robust physiological responses, even with small increases in CO_2_ (26). The phenotypic response to rising CO_2_ levels is to increase minute ventilation by adjusting the depth of breathing [tidal volume (TV)], the frequency of breathing, or both (25–27). Recovery from ventilatory challenges results in a gradual return to baseline minute ventilation as blood levels of CO_2_ decrease and blood levels of oxygen (O_2_) increase (26). Disordered breathing patterns during recovery from environmental stressors indicate respiratory distress and can include periods of abnormally large TVs (sighs) coupled with apneic-like periods of extended pauses between breaths [end-expiratory pause (EEP)]. Large TVs and extended EEPs limit the ability to reduce increased blood levels of CO_2_ by reducing the rate of gas exchange in the lungs (28). As disordered breathing patterns would reduce the ability to appropriately respond to, or recover from, exposure to exogenous stressors including hypercapnia or hypoxia, it has been suggested that such respiratory dysregulation could increase vulnerability to SIDS (29–31).

To determine if loss of cerebellar Purkinje cells results in distressed breathing following exposure to increased environmental CO_2_, we used whole body plethysmography (WBP) to investigate the hypothesis that *Lurcher* mutants heterozygous for the *Lurcher* spontaneous mutation (*Grid2^*Lc*^*), would exhibit disordered breathing patterns during recovery from hypercapnia, in comparison to wild-type (WT) controls. *Lurcher* mice were selected because we had previously observed that deficits in breath frequency appeared during recovery, following exposure to low levels of CO_2_ (24). In the current study, we specifically targeted two components of breath frequency, including TV and EEP periods. These dependent variables were selected because of their importance in modulating increased blood levels of CO_2_ by affecting the rate of gas exchange in the lungs.

## Materials and Methods

### Animals

Mice were bred and housed in the Animal Care Facility of the Department of Psychology at the University of Memphis. They were maintained in a temperature-controlled environment (21 ± 1°C) on a 12:12 light–dark cycle (lights on at 0700 hours) and given free access to food and water. Original *Lurcher* (#001046; Mouse Genome Identifier #: 1857337) breeders were purchased from The Jackson Laboratory (Bar Harbor, ME, USA). All experiments were conducted with strict adherence to the National Institutes of Health Guidelines for the Care and Use of Laboratory Animals. The protocol was approved by the University of Memphis Institutional Animal Care and Use Committee.

The breeding of *Lurcher* mice entailed filial pairing of a non-ataxic female WT (B6CBACa *A^*w-J*^*/*A*-*Grid2*^+^) with a mutant ataxic male heterozygous for the *Lurcher* spontaneous mutation (B6CBACa *A^*w-J*^*/*A*-*Grid2^*Lc*^*). This breeding strategy produced litters that are composed of both heterozygous *Lc/*+ and WT mice. The *Lc/*+ mouse is phenotypically distinguishable from WT littermates as early as postnatal day 12 (PND 12) with cerebellar signs, including an ataxic gait, which permits non-invasive differentiation of *Lc/*+ mice from their non-ataxic WT littermates (32, 33).

Animals were weaned at PND 25 ± 4 days, and sibling housed in groups of three to five in ventilated polystyrene cages. In order to avoid litter effects, six *Lc/*+ and six WT littermates were selected randomly, each pair from different litters and mating cages. All mice were PND 60 at testing onset with a mean weight of 20.05 g (SD = 1.79 g). The subjects consisted of 12 male mice. Male mice were chosen because the incidence of sudden death and cerebellar neuropathology is much larger in males than in females (1, 34).

### Whole Body Plethysmography

Experimental data were collected during light hours using a WBP system (Emka Technologies, Falls Church, VA, USA), as previously described (24). Briefly, a transducer was mounted to the WBP device, which converted pressure differentials in the chamber caused by respiration into electrical signals that were then transmitted to, and interpreted by, the software. Data were collected for each individual breath with sampling rates set at least twice the typical eupneic breathing levels observed in mice: typical TV <30 mL, typical expired volume <30 mL, and typical breath frequency <500/bpm (35, 36), to account for changes in breathing due to the response to, and recovery from hypercapnia. Pressure differentials that met all of these requirements, as interpreted by the software, were counted as individual breaths and added to the data output. Differentials that did not meet all of these parameters were flagged as movement artifacts and not included in the data file for analysis.

### Procedure

Mice were weighed prior to placement in the WBP chamber. The experimental room temperature (22 ± 3°C) and humidity (20 ± 5%) were monitored daily to ensure stability throughout the study. An in-house program coupled to EMKA software was used to assess the subjects’ respiratory responses at baseline (normal room air: 21% O_2_, 0% CO_2_, and 79% N_2_), under conditions of increased CO_2_ (2, 4, 6, and 8%), and during recovery from each CO_2_ challenge in normal room air. Each test day began with a 10-min habituation period, followed by a 4-min exposure to baseline (room air) period. Under all conditions, air in the WBP chamber was removed continuously (0.8–1.0 L/m), to prevent accumulation of excessive CO_2_ due to exhalation.

#### Baseline

After the 10-min habituation period, the dependent variables TV and EEP were continuously recorded, while the mice were exposed to room air for a total of 4 min (21% O_2_, 0% CO_2_, and 79% N).

#### Hypercapnia and Recovery

The entire hypercapnia program was 52 min in duration and consisted of an initial baseline (4 min) measured as described above, followed by four sequential challenges where CO_2_ content was progressively increased from 2, 4, 6, and 8% (21% O_2_, N_2_ on balance). Each of the four CO_2_ challenges consisted of a 2-min chamber fill period (the approximate time required for the WBP to fully achieve the desired gas percentages), followed by a 4-min exposure. To assess the recovery responses to multiple hypercapnic challenges, the program returned to room air following each challenge (again, including a 2-min chamber refill period and a 4-min recovery period). At termination of the final CO_2_ exposure (8%), and return to room air, the mouse was removed from the chamber.

### Variables and Data Analysis

To maintain sample equality among all animals on the measure of breath frequency, the dependent variables, TVs and EEPs, were recorded for the first 510 breaths from each animal during baseline and during recovery from each of the CO_2_ challenge conditions. Because TV and EEP are each components of breath frequency, the breath samples from each animal were made equal at 510 breaths based on the animal with the lowest breaths per minute. Analyses were only conducted on individual breaths to specifically determine if genotypic differences in TVs and EEP durations contributed to previously observed deficits in overall breath frequency during recovery from increased levels of CO_2_.

#### Animal Weights

An independent samples *t*-test was performed with genotype (*Lc*/+ and WT) as the independent variable and weights as the dependent variable to determine whether there was a need to include weight as a covariate, since lung function is tightly correlated with body size (37).

#### Baseline

Baseline data were analyzed using repeated measures analysis of variance (RMANOVA), with genotype (*Lc/*+ and WT) as the between-subjects factor and with the first 510 breaths as the within-subjects factors. Thus, the omnibus analysis was a 2 (Genotype) × 510 (Breaths) mixed design.

#### Hypercapnia Recovery

The recovery conditions were analyzed using an omnibus RMANOVA with genotype (*Lc/*+ and WT) again serving as the between-subjects factor. Within-subjects factors included four levels of recovery from CO_2_ exposure (2, 4, 6, and 8% CO_2_), and from each recovery period, the first 510 breaths to accurately track TVs and moment-to-moment changes in EEP. Therefore, the omnibus analysis became a 2 (Genotype) × 4 (Recovery from CO_2_ exposure) × 510 (Breaths) mixed design. Depending on the results of the omnibus RMANOVAs, additional simple-effects tests were used to analyze interaction effects.

## Results

### Animals

An independent samples *t*-test revealed no significant difference between the body weights of the two genotypes, *t*(10) = −0.497, *p* = 0.630.

### Baseline

**Figure 1:**
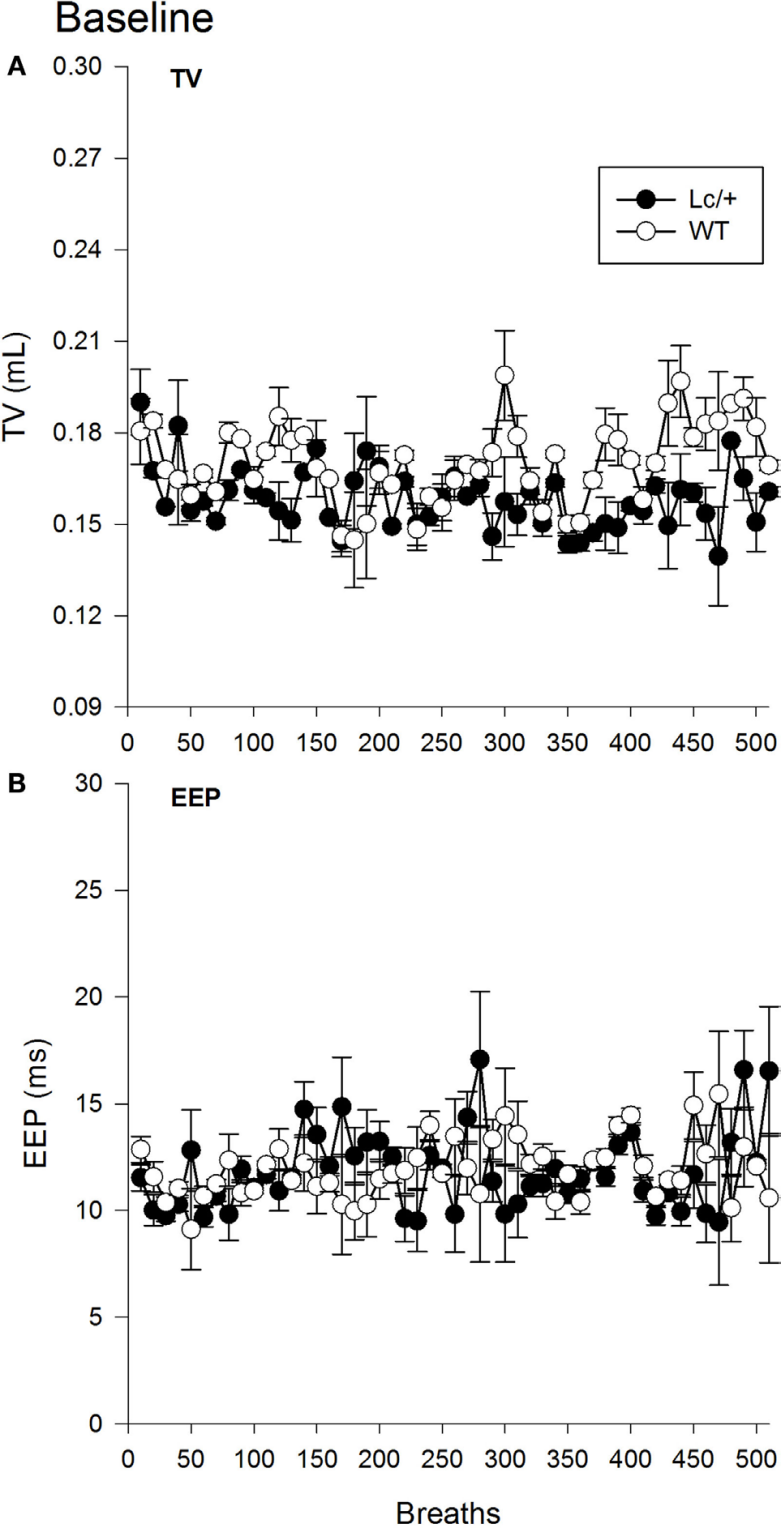
**Baseline tidal volume (A) and end-expiratory pause (B) data for 510 breaths at normal room air in *Lurcher* mutant (*Lc/*+) and wild-type (WT) control mice**. Breaths have been averaged into groups of 10 for clarity of presentation. Values represent mean ± SEM.

The omnibus RMANOVA revealed the depth of breathing (TV) in the two groups was equivalent during normal room air [*Lc/*+ *M* = 0.158 mL, WT *M* = 0.170 ms, SEM 0.013 mL; Group, *F*(1, 10) = 0.879, *p* = 0.371]. Figure 1A shows the mean TVs of the two groups for the first 510 breaths. Note that breaths have been averaged into groups of 10 for clarity of presentation [Breath, *F*(509, 5090) = 0.970, *p* = 0.670; Group × Breaths, *F*(509, 5090) = 0.900, *p* = 0.940].

A second omnibus RMANOVA further revealed the two genotypes had equivalent EEP durations during exposure to room air [*Lc/*+ *M* = 11.819 ms, WT *M* = 11.922 ms, SEM 1.233 ms; Group, *F*(1, 10) = 0.007, *p* = 0.935]. As shown in Figure 1B, the two groups maintained equivalent EEP intervals throughout the continuum of 510 breaths and across individual breaths [Breath, *F*(509, 5090) = 0.891, *p* = 0.957; Group × Breaths, *F*(509, 5090) = 1.011, *p* = 0.427].

### Hypercapnia Recovery

**Figure 2:**
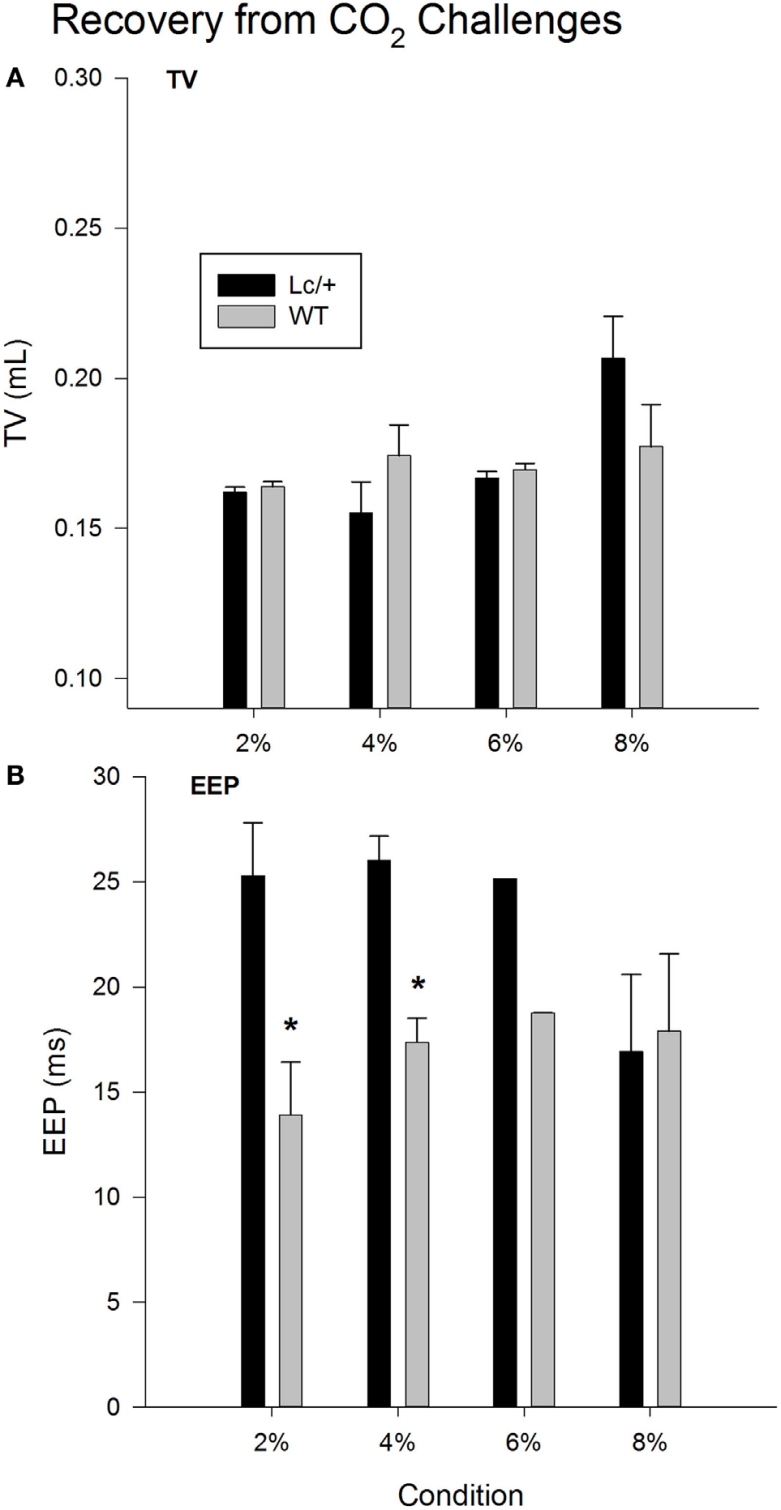
**Recovery tidal volume (A) and end-expiratory pause (B) data, averaged across 510 breaths at room air (21% O_2_, 79% N_2_, and 0% CO_2_), following each CO_2_ exposure period in *Lurcher* mutant (*Lc/*+) and wildtype (WT) mice**. Values represent mean ± SEM. Asterisks indicate significant differences found between genotypes.

Figure 2A shows changes in average TV during recovery following each exposure to CO_2_. Changes in TV were comparable in the two genotypes. Mice in both groups showed a typical increase in TV following exposure to increasing percentages of CO_2_, indicating increased work to reduce the body burden of CO_2_ [2, 4, 6, and 8% CO_2_; Recovery, *F*(3, 30) = 3.765, *p* = 0.021]. Additionally, when considered across the 510 breaths within each recovery condition, both genotypes showed a gradual decline in TV, likely corresponding to gradual reductions in body levels of CO_2_ with the return to room air [data not shown; Breaths, *F*(509, 5090) = 1.420, *p* < 0.001].

As shown in Figure 2B, when comparing recovery conditions, EEPs between the two genotypes differed significantly [Recovery × Group, *F*(3, 30) = 4.044, *p* = 0.016]. Simple-effects tests revealed that *Lc/*+ mice had significantly longer average EEP periods than WT mice during recovery from 2 and 4% CO_2_ [2% Group, *F*(1, 10) = 6.295, *p* = 0.031; 4% Group, *F*(1, 10) = 6.457, *p* = 0.029]. Although genotypic differences only approached significance following exposure to 6% CO_2_ [Group, *F*(1, 10) = 3.128, *p* = 0.107], there was an interval of approximately 100 breaths when *Lc/*+ mice had significantly longer EEPs than WT controls during recovery from 6% CO_2_ [data not shown; Group × Breaths, *F*(509, 5090) = 1.114, *p* = 0.046].

**Figure 3:**
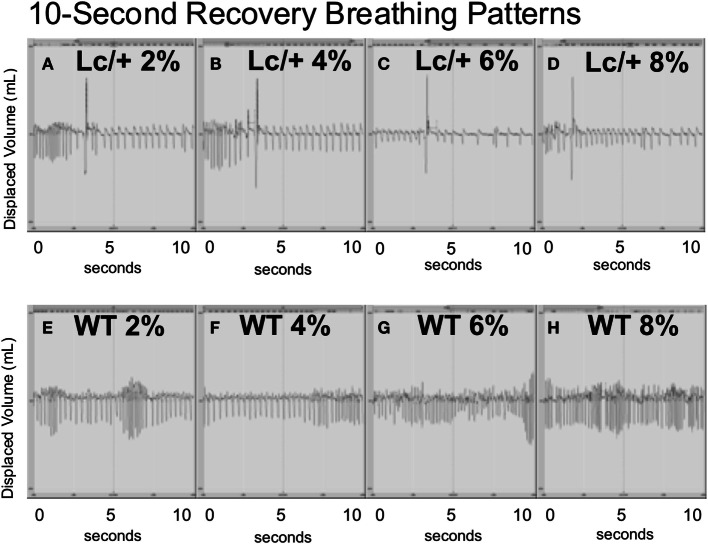
**All data including breaths during 10-s intervals from the middle point of each recovery period (at room air) following exposure to successive CO_2_ challenges (2, 4, 6, and 8%) in an *Lc/*+ mouse (A–D), and a WT control (E–H)**. Data are centered on a zero line on the *y*-axis with inhalations falling below the zero line, and exhalations rising above the zero line. The patterns depicted with the above pair were consistent across all *Lc/*+ mice and wildtype controls.

Figure 3, graphically illustrates the pattern of individual breaths from a representative mutant and WT mouse at the midpoint of each 4-min recovery period following successive CO_2_ exposures. Each panel of this figure includes the entire data form, over 10-s containing approximately 40 breaths. Visually, the two genotypes differed greatly in their breathing patterns. During the recovery periods following CO_2_ exposure, the *Lc/*+ mice exhibited a distinctly abnormal respiratory pattern, visually consistent with previous reports of post-sigh apnea in mice (38). Thus, the *Lc/*+ mouse had abnormally deep breaths (>100% of the average TV over the previous 10-s), which were followed by multiple breaths consisting of atypically long EEPs (>100% of the average over the previous 10-s). The WT mice, however, did not exhibit this pattern of disordered breathing, maintaining a more uniform pattern, including TVs and EEPs that did not differ in volume or length.

## Discussion

*Lc/*+ mice and their WT littermates showed similar patterns of respiration when breathing room air at baseline. This finding further substantiates earlier results, indicating that cerebellar Purkinje cell loss has little effect on components of eupneic breathing (11, 12, 24).

The main outcomes of this study are that developmental loss of cerebellar Purkinje cells significantly reduced the ability of mice to compensate for the increased body burden of CO_2_ as indicated by extended EEP periods following exposure to relatively low levels of hypercapnia. This is important because reduced compensatory responses to the increased body burden of CO_2_ would result in prolonged hypoxemia, which itself could facilitate the cardiorespiratory failure that likely precedes sudden death events (28, 30). A similar pattern of disordered breathing has been documented in a report of sudden onset gasping and apnea in nine infants who subsequently died from SIDS (39). Collectively this preclinical and clinical evidence indicates the importance of evaluating breathing patterns and their potential contribution to impending fatal events. That *Lc/*+ mutants breathing approximated WT levels at 8% further suggests that the threshold for detection for rising levels of CO_2_ in the mutants is much higher than that observed in control mice. This putative deficit in sensitivity to low levels of CO_2_ likely results in a reduced ability to compensate for rising blood levels of CO_2_ and the reduced ability to recover homeostatic ventilatory patterns. Since the cardiovascular system is closely integrated with breathing, disordered cardiovascular patterns, including cerebral blood flow, blood pressure, and heart rate, likely simultaneously occur (25–27). This possibility deserves further investigation.

Mechanisms potentially responsible for the patterns of disordered breathing observed in *Lc/*+ mice are unlikely to include peripheral muscular deficits, as *Lc/*+ mice show more fatigue-resistant diaphragm musculature than WT mice (40). Relatedly, a previous examination of the diaphragm in near-miss sudden death infants also revealed more fatigue-resistant diaphragm muscles compared to controls (41).

A more likely mechanism for the deficits in the recovery patterns of *Lc/*+ mice involves the absence of Purkinje cell input to the FN. The FN are predominately innervated by Purkinje cells of the cerebellum, especially those originating from the vermis, and serve significant roles in cardiorespiratory compensatory responses to multiple challenges, including hypercapnia and hypoxia (8, 10, 42–44). The FN are highly responsive to blood CO_2_ changes, and to a lesser degree, O_2_ changes, and modulate respiration during challenging conditions, but not during normal, rhythmic breathing. Cell body lesions of this structure attenuated responses to increased levels of environmental CO_2_, but had no effect on breathing patterns in room air conditions (10, 14, 23). Structurally, following the loss of 100% of cerebellar Purkinje cells, *Lc/*+ mice exhibit a nearly 60% reduction in FN size, making it reasonable to conclude that this loss of volume could reduce FN-mediated chemosensitivity (45, 46). Such an inability to appropriately detect and respond to increasing CO_2_ levels could increase the risk of a fatal outcome in chemosensitive-related extreme challenges, such as those expected in conditions such as SIDS or postictal periods in epilepsy patients.

Deficits in serotonergic neural systems may also play a role in the current findings. Cerebral serotonin [5-hydroxytryptamine (5-HT)] has been repeatedly implicated as integral in respiratory modulation, and pathophysiological changes in the number or distribution of central 5-HT-containing neurons may play a role in disordered breathing associated with sudden unexplained death (28, 47, 48). *Lc/*+ mice, in comparison to controls, show cerebellar patterns of disordered 5-HT that include a redistribution of 5-HT levels, resulting in increased levels in the deep cerebellar nuclei, including the FN, as well as increased serotonin binding in these nuclei (49, 50). In normal mice, an extensive 5-HT projection to Purkinje cells, originating from medullary and pontine respiratory areas exists (51, 52). As *Lc/*+ mutants lack Purkinje cells, the increased 5-HT projection to the deep cerebellar nuclei possibly represents a reprograming of this serotonergic projection from the Purkinje cells to the deep cerebellar nuclei. This speculative reprograming, in turn, may provide an additional contribution to the loss of sensitivity to low levels of CO_2_. Thus, the differences observed in recovery patterns between *Lc/*+ and WT mice could result from neurochemical events that unfold relative to the developmental Purkinje cell loss in *Lc/*+ mice and further suggest an integral role of the cerebellum in respiratory modulation.

Other disorders also exude the characteristic traits of cerebellar neuropathology, including Purkinje cell loss, shrinkage of deep cerebellar nuclei, serotonergic disruptions, and disordered breathing patterns accompanying unexpected fatalities. Such signs are frequently found in epilepsy. People with epilepsy are roughly 24 times more likely to die unexpectedly than people without epilepsy (53), and ventilatory failure is a major suspect in the fatal event. As in SIDS, SUDEP is an exclusionary diagnosis, and SUDEP victims are typically found in the prone position in bed, which supports the possibility that death has occurred during sleep (54, 55). Postmortem examination of the brains of patients with chronic epilepsy and SUDEP victims consistently reveals cerebellar neuropathology, including cerebellar atrophy and Purkinje cell loss (56–58). Examination of the cerebellum of SUDEP victims revealed significantly higher amounts of Bergmann’s gliosis and folial atrophy compared to age- and sex-matched controls (57). Additionally, decreased cerebellar Purkinje cell densities in either the anterior or posterior lobe appear in SUDEP victims relative to controls (56). Antiepileptic drugs (AEDs), specifically phenytoin (Dilantin), exacerbate cerebellar atrophy and Purkinje cell loss found in patients with epilepsy, and cause cerebellar neuronal damage in both humans and animal models (56, 59–63). Additionally, AEDs, including phenytoin and benzodiazepines, such as clonazepam are associated with an increased incidence of sleep-disordered breathing (64).

Sleep-disordered breathing frequently presents comorbidly with epilepsy (64, 65) and even without treatment with AEDs; patients report excessive daytime sleepiness, which can be an indicator of undiagnosed sleep apnea (66–72). The relevance of these findings for the current study is that sleep-disordered breathing, even without evidence of epilepsy or AEDs, induces significant cerebellar injury (73), and such injury can further intensify disturbed respiratory patterning.

Sudden unexplained death in childhood (SUDC), another exclusionary fatal event, occurs in children >1 year of age (74). It has been speculated that the mechanisms underlying sudden death in SIDS, SUDEP, and SUDC may be closely related (75). Evidence for such a link includes the finding that SUDC victims were significantly more likely to die unexpectedly when one or more febrile seizures were experienced (76). Additionally, an inverse relationship between SIDS and SUDC cases has recently been reported, suggesting that children who die of SUDC may have escaped the window of SIDS only to fail to respond appropriately to a subsequent exogenous stressor (77). As cerebellar neuropathology is often reported in sudden death syndromes, such as SIDS and SUDEP (18, 19, 56–58), and because the cerebellum, especially the Purkinje cells, are particularly vulnerable to hypoxic insult (78), this raises the suspicion that such pathology may be present in the brains of SUDC victims. This possibility, however, has yet to be investigated as research on SUDC is still rudimentary.

Human clinical studies have yielded inconsistent support for the notion that cerebellar neuropathology is involved in sudden death events. For example, in 19 SIDS cases and 12 age-related controls, immunohistochemical staining of the cerebellar vermis revealed evidence of delayed maturation of the cerebellar cortex (18). Further, histological examination at autopsy for 35 cases of unexplained death and 20 controls revealed cerebellar cortex alterations including delayed maturation and apoptosis of the cerebellar Purkinje cells in the sudden unexplained death victims but not the controls (19). Conversely, two separate examinations of the left and right hemispheres of 12 SIDS victims and age- and sex-matched controls revealed no difference between the groups in cerebellar Purkinje cell density (79), Purkinje cell layer volume, or Purkinje cell number (80). However, as cerebellar Purkinje cells are especially vulnerable to even short periods of intermittent hypoxia (78), in the aforementioned studies, the use of controls who had died from conditions involving hypoxia (e.g., suffocation, carbon monoxide intoxication, and heart defect) leaves open the possibility that differences in Purkinje cell number between the two groups were mitigated by circumstances surrounding death. Further, these two studies did not examine the cerebellar vermis – the area of the cerebellum where the Purkinje cells that predominately innervate the FN arise from (81). As the FN is one area responsible for modulating chemosensitive responsivity in situations such as hypoxia (which are believed to occur just prior to unexpected death), it would be of value to include examination of the cerebellar vermis in future studies of sudden unexpected death victims including SIDS.

Collectively, it appears that developmental damage to the cerebellum may play an important role in the development of, or vulnerability to serious, and perhaps life-threatening breathing patterns in several patient populations. Multiple mechanisms may underlie cerebellar Purkinje cell loss, including genetic endowment, excitotoxic injury due to excessive neural activation in epilepsy, treatment with AEDs, hypoxic injury, and surgical insult. Despite various sources of damage to Purkinje cells, the present findings suggest that a potential for impaired breathing patterns exists, including a reduced ability to recover from mild hypercapnia, as demonstrated by a heightened incidence of apneic episodes (i.e., increased EEPs). Several patient populations at risk for sudden death exhibit a combination of cerebellar neuropathology and sleep-disordered breathing, a relationship that suggests that cerebellar injury may contribute to inappropriate responses following hypercapnic exposure that may lead to seriously compromised breathing. The interactions among cerebellar Purkinje cells, their output to autonomic- and respiratory-mediating FN, and integration with other neurotransmitter systems, such as serotonin, that regulate breathing patterns merits continued investigation along with the possibility of sex differences similar to those reported in the human population in sudden death events.

## Author Contributions

MC designed the software program and oversaw data acquisition. JH was integral in data acquisition and provided revision input. RH, DG, and GM conceived this work. GM oversaw data acquisition, and performed analyses and interpretation. GM and MC cleaned data, analyzed and interpreted data, and drafted the manuscript and revisions. RH and DG provided substantial revision guidance. All authors were involved in the final approval of the manuscript and agree to be accountable for all aspects of the work in ensuring that questions related to the accuracy or integrity of any part of the work are appropriately investigated and resolved.

## Conflict of Interest Statement

The authors declare that the research was conducted in the absence of any commercial or financial relationships that could be construed as a potential conflict of interest.
